# Hemiplegia Following Influenza, in a Horse

**Published:** 1881-04

**Authors:** James H. Trink

**Affiliations:** St. John’s, New Brunswick


### V.—HEMIPLEGIA FOLLOWING INFLUENZA, IN A HORSE.

EDITOR JOURNAL OF COMPARATIVE MEDICINE :—

As correspondence and communications are invited from members of the profession, and especially from those who contribute towards the support of your excellent journal, I have to offer your readers a case which occurred in my practice in this city, which was attended with considerable interest to me, and may be, to others.

In the spring of 1880 I was called to see a case reported to be distemper. On approaching the animal, the peculiar appearance of the head struck me at once. Pulse fifty-two, respiration slightly increased; auscultation revealed slight bronchitis. There was a discharge from left nostril, which was sticky and glutinous, much resembling glanders, and adhering around the edge of the nostril. The submaxillary glands were slightly enlarged; bowels somewhat constipated; urine high colored. The ear, eyelid, and all of the nostril of the left side were drooped; the tongue protruding from the side of the mouth; pupil slightly dilated; deglutition was not apparently much affected, but the animal found much difficulty in masticating its food, taking it in its mouth and rolling it out again. Although the animal managed to swallow some, my diagnosis, after examination, was hemiplegia, concomitant with influenza. I had the animal brought out, though with difficulty. It was rather ludicrous to see him walking "side on;" when urged to trot, he fell, and only rose with great difficulty.

I could assign no cause why this lesion should have occurred, save that he had been worked hard at the outset of the disease.

*Treatment.*—Gave half-pint doses of linseed oil until bowels became relaxed, being afraid to give a purgative on account of respiratory organs being affected; rubbed strong ammonia liniment along the course of the spine. Administered potass. iod. in two drachm doses twice a day, which was given for three weeks. At the expiration of that time the influenza had disappeared and with it the hemiplegic symptoms, except the ear, which still drooped.

Mastication had become perfect. At the expiration of six weeks, the animal had gained considerably in flesh and was sent to Boston to be sold, contrary to my advice; for whilst being shipped, amidst the noise and bustle, I noticed cerebral disturbance. As I said before, the ear was slightly drooped, and I was not altogether satisfied that everything was right. However, after being there two days, whilst on the track, he dropped dead. So I was told by the seller. Some one in Boston may have further particulars.

JAMES H. TRINK, V.S.

St. John's, New Brunswick, Feb 16th, 1881.

